# Health Risk Assessment of Heavy Metal Pollution in Groundwater Around an Exposed Dumpsite in Southwestern Nigeria

**DOI:** 10.5696/2156-9614-9.24.191210

**Published:** 2019-12-06

**Authors:** Temitope A. Laniyan, Adeniyi J. Adewumi

**Affiliations:** 1 Department of Environmental Health Sciences, College of Medicine, University of Ibadan, Ibadan, Nigeria; 2 Department of Geological Sciences, Achievers University, Owo, Ondo State, Nigeria

**Keywords:** dumpsite, leachates, corroded, groundwater, indecisive, health risk

## Abstract

**Background.:**

Groundwater quality can be poor in Nigeria due to indiscriminate refuse dumping. Exposed dumps serve as point source pollution that discharge potentially toxic heavy metals into the environment.

**Objectives.:**

The present research aimed to assess the impact of metal pollution on groundwater quality in hand-dug wells around an active dumpsite and to evaluate the long-term human health effects of this pollution.

**Methods.:**

Water samples from hand-dug wells used for drinking, irrigation and domestic purposes were collected around the dumpsite. Two samples were collected at each location for cation and anion analyses. Samples for cation analysis were acidified with concentrated hydrochloric acid to preserve the elements in the samples, while those for anion analysis were not acidified. Collected samples were analyzed using inductively coupled plasma mass spectrometry and atomic absorption spectroscopy.

**Results.:**

Mean concentrations of metals and physical parameters were compared with the World Health Organization's standards (2012). All samples were found to be within permissible limits, except for arsenic (As) (0.13 mg/L), potassium (K) (29.94 mg/L), lead (Pb) (0.38 mg/L), cadmium (Cd) (0.003 mg/L) and average temperature (31.93°C) as a result of corroded service pipes containing Pb in the dumpsites and the reaction of leachates with various materials such as used battery, tins, and electronic wastes which later leaked into the groundwater. The geoaccumulation index revealed Pb to be moderately to highly contaminated in groundwater.

**Conclusions.:**

Heavy metal pollution can cause deleterious health effects that can lead to short- and long-term diseases such as keratosis (skin hardening), lung cancer, bladder cancer and ultimately death if proactive steps are not taken. Disposal bags should be provided to all houses in the area, as well as guaranteed waste disposal trucks and dispose of waste at approved sites. In addition, enforcement agents should ensure compliance with rules and regulations. A centralized, deep, double-cased well should be constructed in a clean environment in the study area for drinking and domestic use.

**Competing Interests.:**

The authors declare no competing financial interests.

## Introduction

Urbanization presents many challenges for developing countries, such as industrialization and over-population which can lead to indiscriminate dumping of waste. Haphazard dumping of domestic garbage, wood, polythene bags, plastics, broken glass, abandoned automobiles, ash, dust, demolition, agricultural, industrial, hospital, and human and animal waste can result in refuse in the environment. Refuse waste becomes a form of point source pollution if it affects the environment directly and non-point source pollution if it affects the environment indirectly. This leads to contamination of surface and groundwater and adversely impacts public health. Nigeria is a lower middle-income country which is facing a myriad of pollution and human and environmental healthissues.^1–10^

Diseases which may lead to increased mortality are relative to gradual increases in arsenic (As), lead (Pb) and cadmium (Cd) in the environment originating from toxic waste in solid or liquid forms.^11–14^ Indiscriminate dumping of refuse has consequences on the environment, such as flooding due to waste blockage of water run-off channels, and emission of contaminants that leads to slight poisoning and eventually highly toxic waters (ground and surface) and may lead to diseases such as asbestosis, respiratory problems, cancer and eventually death.^5^,^15–17^ Studies have shown that dumpsites consist of metals, nylon, cans, syringes, electronic and animal wastes, municipal, commercial, hospital and mixed industrial wastes, threaten the quality of groundwater, which is the main source of drinking water for the surrounding community.^7^,^18–21^

Many studies have been conducted on the effects of heavy metals on groundwater around dumpsites, but few have investigated the long-term health risk of metals such as Pb, As and Cd on children and adults.^5^,^11^,^12^,^16^,^17^ The present study will help to fill this gap with findings on health risks due to indiscriminate dumping of refuse.

The present study aimed to assess the impact of metal pollution on groundwater quality in hand-dug wells around an active dumpsite and to evaluate the long-term effects of this pollution on public health.

## Methods

A geochemical survey of metals in the groundwater around the exposed dumpsite was conducted to determine the impact on the environment. The groundwater in the study area was used for drinking and domestic purposes.

### Study area

**Figure 1:**
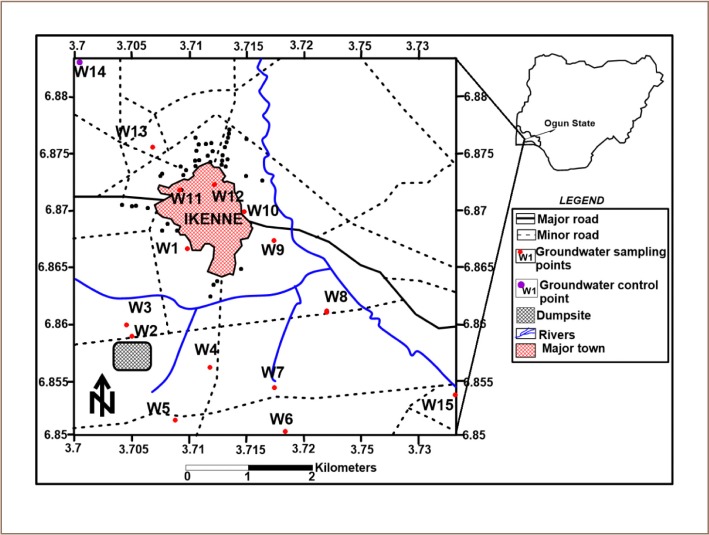
Location map with sampling points

The study area lies within latitude 06°51′00″ N - 06°53′00″ N and longitude 003°42′00″ E - 003°44′00″ E *(Figure 1)*. The area has a moderately high relief; it is easily accessible with a series of interconnecting minor roads, footpaths and a few major roads. The drainage pattern is dendritic, showing irregularity in direction. The climate is characterized by a tropical climate with alternating wet and dry seasons which gives the study area typical rain forest vegetation. Ikenne is part of the southwestern Nigerian basement complex terrain; it is representative of both a migmatite gneiss complex and older granite, which shows structural disposition.^22^ The migmatite gneiss complex occupies the southwestern and southeastern part of the study area, while older granite occupies the north, east and a portion of the western area.^23^ Rock found in the area occurs directly above or is covered by a shallow mantle of superficial deposits. The complex is an extension of the Togo – Benin – Nigeria shield which occurs east of the West African craton.

**Abbreviations**
*C F*: Contamination factor
*EC*: Electrical conductivity
*EF*: Enrichment factor
*Igeo*: Geoaccumulation index
*TDS*: Total dissolved solids
*WHO*: World Health Organization

The study area is located at Aiyepe/Ikenne, with the headquarters in Ikenne. Ikenne is located between Sagamu and Odogbolu, and is very close to Ilisan-Remo, southwestern Nigeria. Ikenne has an area of 144 km^2^ and had a population of 118735 in the 2006 census.^24^ The climate and geographical location supports a wide range of economic activities such as agriculture, industrial and commercial activities. The town is 11.7 km from Sagamu and has a boundary between Odogbolu and Ilisan.

Ikenne harbors a 20-year-old dumpsite that is almost an acre across. The dump is situated along the Ikenne-Aiyepe road. The Sagamu-Benin Express Road is the dividing line between Ikenne and Ilisan. The dumpsite serves as the main location for waste disposal for Ikenne and the surrounding area. Presently, however, the dumpsite poses a major threat to soils and human health, as it is located within a large community with many farms and hand-dug wells. During the rainy season leachates from the dumpsite are transported and accumulated in groundwater. Various wastes such as vegetable matter, domestic wastes, human and animal wastes, organic and inorganic matter, wood, paper, cloth, glass, plastic of various types, unclassified metal scraps, empty cans of various chemicals, and other household trash are some of the refuse that is found on the dumpsite that is operated as an open dump with intermittent open-air incineration.

### Sampling and analysis

Sampling was carried out in June 2016; all the instruments used in the field were cleaned with de-ionized water before use. A clean measuring tape attached to the base of a clean stone was used to determine the depth from the water surface to the wellhead and measurements were recorded at each location. A new, clean fetcher was used to draw water samples from each well and global positioning system was used to coordinate each location. A control sample was taken from groundwater in a relatively clean area. The samples were collected 20 m away from the dumpsite. Two samples were chosen at each location for cation and anion analysis, those chosen for cation analysis were acidified with concentrated hydrochloric acid to preserve the elements in the samples, while those chosen for anion analysis were not acidified. Total dissolved solids (TDS), pH, salinity and temperature were measured in situ, and all collected samples were sent for analysis at the Redeemer's University, Ede, Osun State, Nigeria.

### Statistical evaluation

Assessment of contamination according to metal enrichment or the enrichment factor (EF) proposed by Sinex and Helz was employed to assess the degree of contamination and to understand the distribution of the elements of anthropogenic origin from sites via individual elements in sediments.^25^ Iron (Fe) was chosen as the normalizing element for determining EF values, as it is one of the widely used reference elements.^26–30^ Other widely used reference metals or elements included aluminum (Al) and manganese (Mn).^31^,^32^ The formula for EF and its classifications are stated in Equation 1:

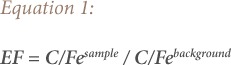
Where, C is the element concentration. In this case, the background value is the concentration of groundwater in the control site and is categorized as ≤ 1, background concentration; 1–2, depletion to minimal enrichment; 2–5, moderate enrichment; 5–20, significant enrichment; 20–40, very high enrichment; and ≥ 40, extremely high enrichment.^28^

### Contamination factor

Contamination factor (CF) was used to determine the contamination status of the groundwater. The formula for the contamination factor in Equation 2:

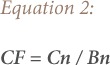



Where, Cn is the concentration of heavy metals in groundwater, and Bn is the background concentration at the control site.

The following categories are suggested for classifying the CF: CF<1, low contamination factor;1 ≤ CF< 3, moderate contamination;3 ≤ CF< 6, considerable contamination factor; CF ≥ 6, very high contamination factor.^30^,^31^

### Geoaccumulation index

As proposed by Muller, the geoaccumulation index (Igeo) has been widely used to evaluate the degree of heavy metal contamination in terrestrial and aquatic environments.^32^,^33^ Igeo is expressed in Equation 3.

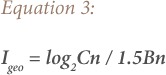



Where, Cn is the concentration of the examined metal in the groundwater, Bn is the background concentration of a given metal, and 1.5 is a factor accounting for possible variation in the background concentration due to lithologic differences. The geoaccumulation index is classified into seven descriptive classes as follows: ≤ 0, practically uncontaminated; 0–1, uncontaminated to slightly contaminated; 2–3, moderately to highly contaminated; 4–5, highly to very strongly contaminated; and ≥5, very strongly contaminated.

### Risk assessment

Risk assessment is used to estimate the impact of carcinogens and the future rate at which an individual is affected by carcinogenic metals. The risk (Risk _pathway_) is the probability of an individual developing cancer resulting from exposure to the potential carcinogenic metal. A Possible pathway includes ingestion, inhalation or dermal contact (*Equation 4*).

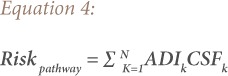



Where, Risk _pathway_ is the unit less probability of an individual developing cancer over a lifetime; ADI_k_ (mg/kg/day) is the mean daily intake; CSF_k_ (mg/kg/day)^−1^ is the cancer slope factor; k is the k^th^ metal; and n is the number of metals.

The human health risk is then calculated by the addition of all results from the three pathways which is the results of risk in inhalation, ingestion and dermal contact (*Equation 5)*:

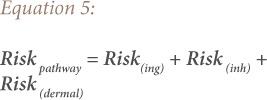



The results of exposure were assessed using Table 1.

**Table 1:** Reference Doses (RfD) in (mg/kg-day) and Cancer Slope Factors (CSF) for the Pathways

**Heavy Metal**	**Oral *RfD***	**Dermal *RfD***	**Inhalation *RfD***	**Oral *CSF***	**Dermal *CSF***	**Inhalation *CSF***	**References**
Pb	3.60E-03	-	-	8.50E-03	-	4.20E-02	34,35
Chromium (VI)	3.00E-03	-	3.00E-05	5.00E-01	-	4.10E+01	36,34
Cobalt	2.00E-02	5.70E-06	5.70E-06	-	-	9.80E+00	36
Nickel	2.00E-02	5.60E-03	-	-	-	-	34
Copper	0.037	0.037	-	-	-	-	34,36
Zinc	3.00E-01	7.50E-02	-	-	-	-	34,36

Abbreviations: R*f*D, reference dose; CSF, cancer slope factor

## Results

The mean concentrations of metals and physical parameters were compared with the World Health Organization (WHO) standard limits in Table 2.^37^ All the samples were found to be within the WHO maximum permissible limits of drinking water except for As (0.13 mg/L), potassium (K) (29.94 mg/L), Pb (0.38 mg/L), Cd (0.003 mg/L) and average temperature (31.93°C).

**Table 2:** Mean Geochemical Values and Physical Parameters of the Groundwater of the Study Area (n = 15) (mg/L)

**Elements**	**Mean concentration**	**WHO (2012)**	**T°C**	**pH**	**EC**	**TDS**	**Depth to water table (m)**
Cu	1.22	2	**32**	8.3	0.5	0.2	27.6
Cd	0.003	0.003	**32**	8.7	0.3	0.1	37.4
As	**0.13**	0.01	**34**	9.2	0.2	0.1	42.4
Pb	**0.38**	0.01	**33**	7.5	0.4	0.2	57.7
Zinc	10.8	75–200	**30**	8.2	0.1	0.1	34.5
Fe	0.05	0.3	**32**	9.2	0.4	0.7	23.3
Ca^2+^	8.87	75–200	**31**	8.7	0.06	0.3	36.3
Mg^2+^	10.43	150	**33**	8.4	0.4	0.2	23.6
Na^+^	10.8	200	**34**	8	1.3	0.6	8
K^+^	**29.94**	12	**35**	8.1	1	0.5	28.9
SO_4_^2−^	6.88	400	**32**	8.8	0.4	0.2	12
NO^3−^	7.15	50	**34**	8.2	0.3	0.1	52
Cl^−^	25.32	250	**30**	7.9	0.3	0.1	64.9
CO_3_^2−^	104	240	**29**	9	0.1	0	51.9
HCO_3_	278.57	500	**28**	9	0.7	0.3	18.4
WHO^35^			27	6.5–9.2	300	500	

Abbreviations: Ca^2+^, calcium(2+), Cu, copper; Mg^2+^, magnesium(2+); Na^+^, sodium(1+); SO_4_^2−^, sulfate(2−); NO^3−^ nitrate(1−); Cl^−^, chlorine(l-); CO_3_^2−^,carbonate; HCO_3_,bicarbonate; T°C, temperature; EC, electrical conductivity; WHO, World Health Organization.

### Contamination assessment of groundwater

The EF of background concentrations for calcium (Ca), magnesium (Mg), sodium (Na), K, copper (Cu), Cd, As, Pb, and zinc (Zn) were found in 8, 6, 1, 6, 7, 8, 5, 6 and 10 samples, respectively. Depletion to minimal enrichment for Ca, Mg, Na, K, Cu, Cd, Fe, As, Pb and Zn were found in 5, 7, 5, 4, 6, 4, 15, 8, 7 and 3 samples, respectively. Moderate enrichment for Na, K, and Cd were found in 6, 3, and 1 sample, respectively. Significant enrichment was found with regard to Ca, Mg, Na, K, Cu, Cd, As, Pb and Zn. Very high enrichment was observed for 2 samples in Na *(Table 3).*

**Table 3:** Contamination Assessment of Groundwater

**Indices**	**Classification**	**Ca**	**Mg**	**Na**	**K**	**Cu**	**Cd**	**Fe**	**As**	**Pb**	**Zn**
Enrichment factor	<1: Background concentration	8	6	1	6	7	8	-	5	6	10
	1–2: Depletion to minimal enrichment	5	7	5	4	6	4	15	8	7	3
	2–5: Moderate enrichment	-	-	6	3	-	1	-	-	-	-
	5–20: Significant enrichment	2	2	1	2	2	2	-	2	2	2
	20–40: Very high enrichment	-	-	2	-	-	-	-	-	-	-
	>40: Extreme enrichment	-	-	-	-	-	-	-	-	-	-
Contamination factor	CF < 1:Low contamination factor	11	1	-	3	2	8	6	1	2	14
	1 < CF < 3: Moderate contamination factor	4	14	12	11	13	7	9	14	11	1
	3 ≤ CF ≤ 6 Considerable contamination factor	-	-	3	1	-	-	-	-	-	-
	CF ≥ 6: Very high contamination factor	-	-	-	-	-	-	-	-	1	-
Igeo	< 0: Practically uncontaminated	15	15	3	1	15	13	14	13	-	15
	0–1: Uncontaminated to slightly contaminated	-	-	12	14	-	2	1	2	14	-
	2–3: Moderately to highly contaminated	-	-	-	-	-	-	-	-	1	-
	4–5: Contaminated	-	-	-	-	-	-	-	-	-	-
	>5: Very strongly contaminated	-	-	-	-	-	-	-	-	-	-

**Table 3:** Contamination Assessment of Groundwater

	**Component**						

	**1**	**2**	**3**	**4**	**5**	**6**	**7**
Temp.	**0.751**	0.123	**0.433**	−0.135	0.243	−0.208	−0.025
Salinity	**0.567**	**0.504**	0.064	**0.488**	0.102	0.082	−0.116
TDS	**0.440**	**0.616**	−0.150	0.092	**0.424**	0.266	0.207
pH	−0.044	0.027	**−0.477**	**0.814**	−0.099	−0.087	0.028
EC	**0.590**	**0.462**	0.137	−0.215	−0.100	**0.466**	0.214
SO_4_	−0.390	**0.402**	0.388	**0.438**	−0.381	0.324	0.189
NO_3_	**−0.493**	**0.437**	0.028	−0.225	**−0.437**	0.371	0.376
Cl	0.150	**−0.522**	−0.298	0.132	0.218	−0.055	**0.606**
CO_3_	**−0.644**	0.065	0.343	−0.224	**0.602**	0.180	−0.021
HCO_3_	**−0.669**	0.143	0.360	−0.094	**0.534**	0.194	−0.207
Ca	0.130	**0.586**	**−0.580**	−0.327	−0.253	0.100	−0.252
Mg	**0.434**	0.167	**0.529**	−0.199	0.217	−0.090	0.387
Na	**0.612**	**−0.588**	**0.422**	0.095	−0.168	0.146	−0.092
K	0.104	**−0.629**	0.046	0.314	0.140	**0.408**	−0.028
Cu	**0.420**	−0.150	**−0.667**	−0.502	−0.094	0.109	0.025
Cd	**−0.536**	**−0.675**	−0.153	0.195	0.004	0.146	0.199
Fe	−0.054	**0.618**	**0.494**	**0.450**	−0.187	−0.157	−0.105
As	−0.250	0.062	**0.462**	−0.234	−0.331	**−0.571**	0.287
Pb	0.128	**0.422**	**−0.473**	0.322	**0.418**	−0.286	0.200
Zn	**0.6**12	**−0.588**	**0.422**	0.095	−0.168	0.146	−0.092
Total	4.220	4.011	3.071	2.206	1.815	1.354	1.094
% Variance	21.10	20.05	15.35	11.03	9.08	6.771	5.472

Abbreviation: Temp., temperature.

### Contamination factor

Results of the contamination factor analysis show that a low contamination factor was observed in Ca, Mg, Na, K, Cu, Cd, Fe, As, Pb and Zn in 11, 1, 0, 3, 2, 8, 6, 1, 2, 1and 14 samples, respectively, while a moderate contamination factor for Ca, Mg, Na, K, Cu, Cd, Fe, As, Pb and Zn was observed in 4, 14, 12, 11, 13, 7, 9, 14, 11 and 1 samples, respectively. Considerable contamination factor was found in Na and K in 3 and 1 samples, respectively.

Only Pb was found to have high degree contamination factor. The degree of contamination in groundwater in the study area was found to have a moderate degree of contamination *(Table 3).*

### Geoaccumulation index

The Igeo results showed Ca, Mg, Cu and Zn to be practically uncontaminated, while Na, K, Cd, Fe and As ranged from practically uncontaminated to slightly contaminated. Only Pb was moderately to highly contaminated in the sampled groundwater *(Table 3).*

## Discussion

The mean Fe^+^ and Cd values (0.05 and 0.003 mg/L, respectively) in the groundwater indicate that most metals did not originate from the weathering of silicate-bearing rocks underlying the area, and thus are from anthropogenic activities (dumpsites) close to the well. Zinc, Cu, Pb, As, and Cd had mean values of 10.80, 1.22, 0.38, 0.130, and 0.003 mg/L, respectively. The concentration of metals in the groundwater in the area occurred in the order of Zn =10.80 > Cu = 1.22 > Pb = 0.38 > As = 0.13 > Fe = 0.05> Cd = 0.003. All of the wells in the study area had values for Pb > 0.2 mg/L, K>12 mg/L, while some had concentrations of Cd> 0.1, which is higher than the WHO permissible limits.^37^ Elevated amounts of Pb may have resulted from batteries and lead pipes in the dumpsite, as when leachates becomes acidic, the acid is released into the groundwater.^38^ Cadmium could be linked with the dispersal of heavy metals produced from electronic wastes disposed of in the dumpsite. High values of K may be linked to leachates, which were also affirmed in the spatial distribution of metals in groundwater in the area (*Figures 2a and 2b*). The general trend showed higher metal pollution in the wells close to the dumpsite and decreased with distance from the dumpsite.

**Figure 2a:**
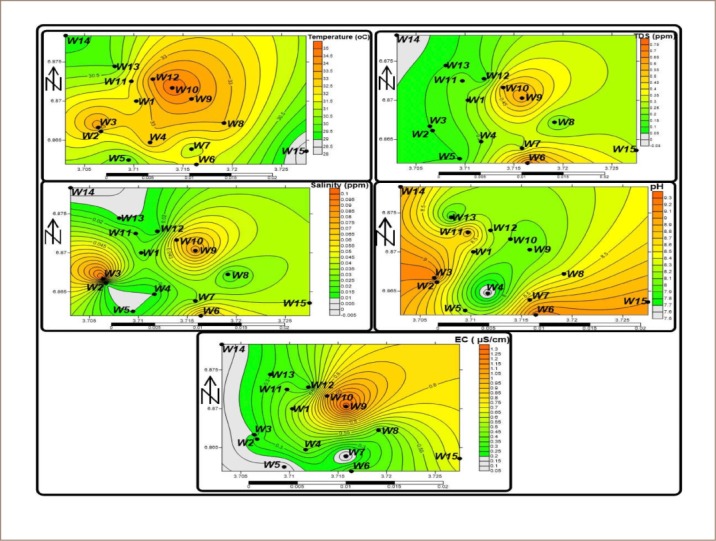
Spatial maps of physical parameters in groundwater of the study area Abbreviations: W, water samples; EC, electrical conductivity.

**Figure 2b:**
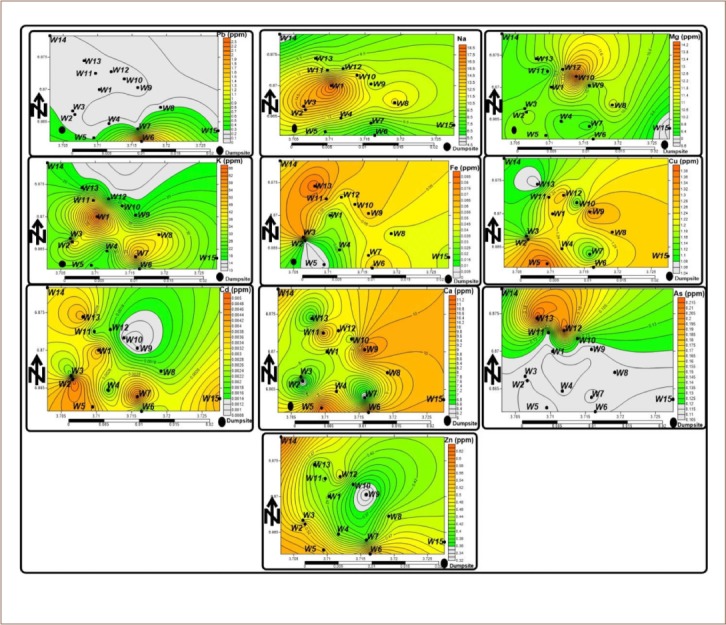
Spatial maps of metals in groundwater of the study area Abbreviations: W, water samples.

### Hydrochemical results of the anion

The high abundance of bicarbonate (HCO_3_) and carbonate (CO_3_^2−^) may be caused by discarded carbonate rich products in the dumpsites and underlying rocks, where interaction between rock and water occur. The high presence of chlorine (1−) (Cl^−^) and sodium (1+) (Na^+^) was attributed to domestic effluents, fertilizers, septic tanks and leachates from sewage in the waste dumpsite.^39^ Low Fe^+^ concentrations revealed an oxidizing environment that is attributed to the oxidation of ferrous to the ferric form.

### Groundwater classification

**Figure 3:**
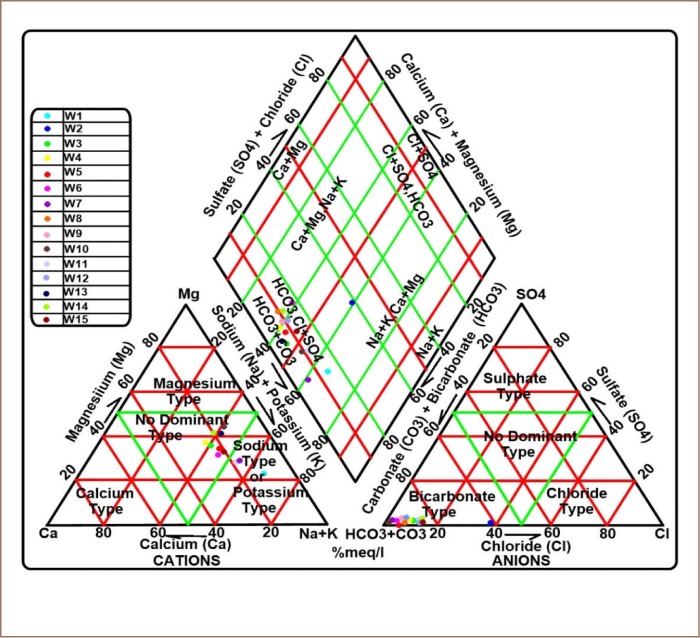
Piper diagram of the study area Abbreviations: W, water samples.

A Piper diagram *(Figure 3)* illustrates the hydrochemical evolution of groundwater by plotting the cations and anions. The Piper diagram consists of three major fields: the cation ternary (left), anion ternary (right) and diamond fields (center). The groundwater samples were plotted on the Piper diagram to determine water types in the area. The plot indicates that 93% of groundwater samples in the study area fall within the (HCO_3_, Cl + sulfate (SO_4_)) − (HCO_3_+carbon trioxide (CO_3_)) groundwater type, while 3% fall into (Na+K) − (calcium (Ca) + magnesium (Mg)) water type. Dumpsites, landfill leachates, and sewages are major sources of bicarbonates in groundwater.^40^

### Bivariate correlation analyses

Bivariate analysis *(Supplemental Material)* between physicochemical parameters revealed strong a relationship (r=0.634) between TDS and salinity. This implies that the overall salinity in groundwater may have originated from the same sources. This was also reflected by the relationships between salinity and other parameters, SO_4_ (r = 0.516), Cl (r = 0.597), HCO_3_ (r = 0.501), Ca (r = 0.621), Mg (r = 0.520), Na (r = 0.603), Cu (r = 0.763) and Zn (r = 0.603). The association of salinity with SO_4_, Cl, HCO_3_, Ca and Mg reflects possible anthropogenic influence and may come from the dumpsite; it could also come from the use of agricultural nitrogen (N), phosphorus (P) and K fertilizers and recycled irrigation water. Total dissolved solvents had an extremely strong relationship with pH (r = 0.915), revealing that TDS determines the degree of acidity and alkalinity in the groundwater of the study area. Similar relationships were found with TDS and SO_4_ (r = 0.911), TDS and nitrate (NO_3_) (r = 0.994), TDS and Cl (r = 0.761), TDS and CO_3_ (r = 0.899), and TDS and HCO_3_ (r = 0.874). The relationship between TDS with NO_3_ indicates pollution from sewage wastes, domestic effluents, and it also suggests the presence of bicarbonate. Chloride and nitrates strongly influenced TDS and electrical conductivity (EC) in the study area. Sulfate also had a strong relationship with NO_3_ (r = 0.650), CO_3_ (r = 0.606), Ca (r = 0.838), Mg (r = 0.828), Na (r = 0.550), K (r = 0.797), Cd (r = 0.953), As (r = 0.608), Pb (r = 0.665) and Zn (r = 0.550), which reflects a possible anthropogenic source. A very strong correlation was noticed between CO_3_ and HCO_3_(r = 0.907), which indicates chemical interactions between rainwater and the aquifer as the former percolates the subsurface. Strong relationships between Pb with Cu (r = 0.721), Cd (r = 0.544), Fe (r = 0.613) and As (r = 0.633) reflect the contribution of heavy metals from the nearby dumpsite.

### Factor analysis

Factor analysis is another form of correlation that helps to describe the possible source of pollution in groundwater. Seven components were extracted for analysis *(Table 4).* Component 1 revealed that the parameters have a common origin which is possibly anthropogenic in nature. Component 2 showed the pollution could originate from agricultural practices and activities at the dumpsite. Component 3 reflects an anthropogenic source, possibly from various wastes discarded in the dumpsites. This component shows that pH-Ca-Cu-Pb has had a negative impact and their presence in the groundwater may be hazardous to human health. In component 4, salinity-pH-SO_4_-Fe are strongly related and may have originated from either a natural or anthropogenic source of contamination. In component 5, associations reflect an anthropogenic source of groundwater contamination. The presence of NO_3_ shows a possible agricultural source, while the presence of Pb shows possible contamination from the dumpsite. In component 6, EC-K-As formed an association revealing an anthropogenic source of contamination from the dumpsite.

### Hierarchical cluster analysis

**Figure 4:**
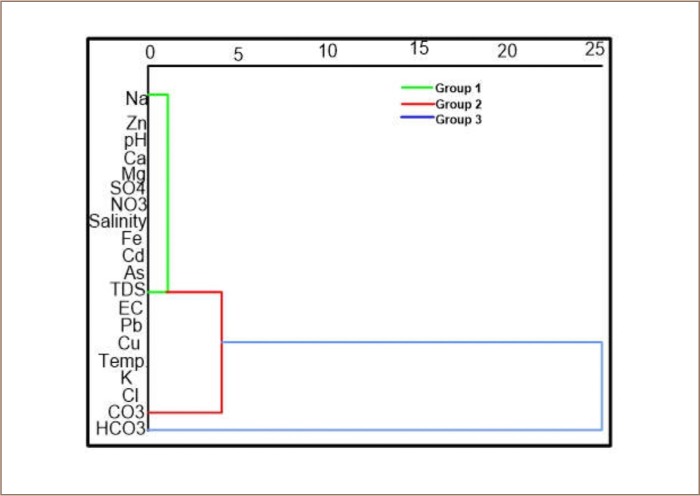
Hierarchical cluster analysis

The dendrogram *(Figure 4)* classifies parameters in the groundwater into 3 groups. In group 1, Na, Zn, pH, Ca, Mg, SO_4_, NO_3_, salinity, Fe, Cd, As, and TDS have a common source of contamination. This may be linked to anthropogenic sources which may be from agricultural practices in the area or from the dumpsite. Group 2 includes K, Cl, CO_3_, TDS, EC, Pb, Cu, and temperature and group 3 consists of HCO_3_, Cu, temperature, K, Cl, and CO_3_. These groupings reflect a possibly natural source of contamination arising from rock-water interactions in the area.

### Evaluation of groundwater for irrigation purposes

Irrigation water indices such as EC, TDS, soluble salt percentage, sodium adsorption ratio, magnesium adsorption ratio and Kelly ratio were used to assess the suitability of groundwater in the area for irrigation purposes.

### Salinity

To determine the amount of dissolved ionic salts present in the groundwater, EC was used, which also measures salinity hazard to crops, as it affects the TDS in groundwater.^41^ The results showed that EC and TDS in all of the groundwater samples was lower than 250 and 1000 mg/l, respectively, and groundwater is therefore safe for irrigation purposes. Soluble salt percentage also evaluates the sodium hazard and was calculated using Equation 6:^42^

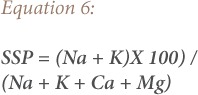



Where, SSP is the soluble salt percentage. The results revealed that 33% of the samples are permissible for use in irrigation, 60% are of uncertain suitability, and 7% of the groundwater samples were unsuitable for irrigation. Sodium adsorption ratio is the best parameter to determine the suitability of groundwater for irrigation, as it evaluates the alkali/sodium hazards in crops.^43^ Sodium adsorption ratio can be measured using Equation 7, as characterized by Karanth.^44^

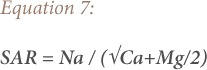



The results revealed that 100% of groundwater samples were suitable for irrigation. The magnesium adsorption ratio was calculated using Equation 8.^45^

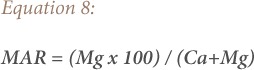



The findings showed that 47% of the samples were suitable for irrigation while 53% were unsuitable. The Kelly ratio values were calculated using Equation 9.^46^

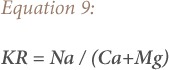



Where, KR is the Kelly ratio. Kelly ratio values <1 showed suitability, while KR > 1 showed unsuitability for irrigation purposes. All of the samples were safe for irrigation *(Table 5).*

**Table 5:** Irrigation Parameters of Groundwater in the Study Area

**Parameters**	**Range**	**Class**	**Number of Samples**	**% of Samples**
EC	<250	Excellent	15	100
	250–750	Good	0	0
	750–2000	Permissible	0	0
	2000–3000	Doubtful	0	0
	>3000	Unsuitable	0	0
TDS	< 1000	Fresh	15	100
	1000–10000	Brackish	0	0
	10000–100000	Saline	0	0
	>100000	Brine	0	0
SSP	<20	Excellent	0	0
	20–40	Good	0	0
	40–60	Permissible	5	33
	60–80	Doubtful	9	60
	>80	Unsuitable	1	7
SAR	0–10	Excellent	15	100
	10–18	Good	0	0
	18–26	Permissible	0	0
	>26	Doubtful	0	0
MAR	≤50	Suitable	7	47
	>50	Unsuitable	8	53
KR	> 1	Unsafe	0	0
	< 1	Safe	15	100

Abbreviations: SSP, soluble salt percentage; SAR, sodium adsorption ratio; MAR, magnesium adsorption ratio; KR, Kelly ratio.

### Health risk assessment

**Figure 5:**
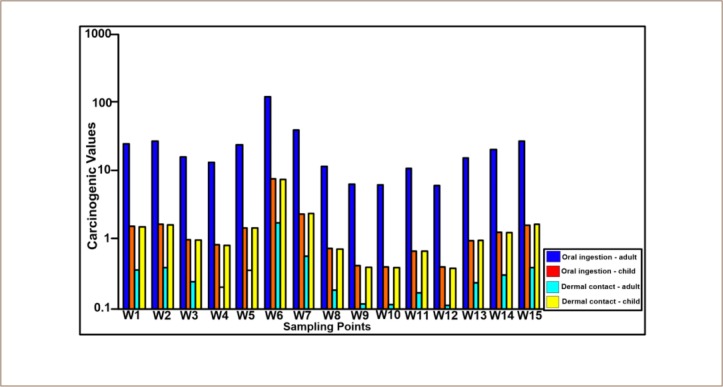
Total carcinogenic health index of heavy metals through oral and dermal contact in water samples from Ikenne Abbreviation: W, sampling point

Health risks in adults and children *(Table 6)* for Pb and As revealed that Pb represents a high carcinogenic health hazard in the area, through oral or dermal contact, as the values of the health hazards are above 10^−4^ and 10^−8^, which is the acceptable range. Use of groundwater for drinking and bathing in this area for long periods of time poses a risk for the development of carcinogenic diseases *(Figure 5).*

**Table 6:** Carcinogenic Health Hazard for Adults and Children through Oral Ingestion and Dermal Contact with Water

	**Oral Ingestion**			**Dermal**			**Hazard index**

	**HQ As**	**HQ Pb**	**HI**	**HQ As**	**HQ Pb**	**HI**	
	
Adults	0.000184	24.11761	24.11779	0.005157	0.344537	0.349694	48.93497
	0.000158	26.09586	26.09602	0.004421	0.372798	0.377219	52.94647
	0.00033	15.39639	15.39672	0.009246	0.219949	0.229195	31.25184
	0.000352	12.81906	12.81941	0.009867	0.183129	0.192996	26.02481
	0.000217	23.3136	23.31381	0.006061	0.333051	0.339112	47.30585
	0.000365	118.5775	118.5778	0.01022	1.693964	1.704184	240.564
	0.000139	37.68786	37.688	0.00389	0.538398	0.542288	76.46058
	0.000378	11.30174	11.30212	0.010582	0.161453	0.172036	22.9483
	0.000787	6.171382	6.172169	0.022038	0.088163	0.110201	12.56474
	0.000729	6.044552	6.045281	0.020399	0.086351	0.10675	12.30406
	0.000375	10.53467	10.53505	0.010499	0.150495	0.160994	21.39208
	0.000722	5.916313	5.917035	0.020215	0.084519	0.104734	12.04354
	0.000299	14.85603	14.85633	0.008371	0.212229	0.2206	30.15386
	0.000213	19.6236	19.62381	0.00596	0.280337	0.286297	39.82021
	0.000179	26.00398	26.00415	0.005006	0.371485	0.376492	52.76129
Children	0.005157	1.464283	1.46944	0.00011	1.464283	1.464393	2.933833
	0.004421	1.584391	1.588812	9.44E-05	1.584391	1.584486	3.173298
	0.009246	0.934781	0.944027	0.000197	0.934781	0.934978	1.879005
	0.009867	0.7783	0.788166	0.000211	0.7783	0.778511	1.566677
	0.006061	1.415468	1.421529	0.000129	1.415468	1.415598	2.837127
	0.01022	7.199345	7.209565	0.000218	7.199345	7.199563	14.40913
	0.00389	2.288192	2.292081	8.30E-05	2.288192	2.288275	4.580356
	0.010582	0.686177	0.696759	0.000226	0.686177	0.686403	1.383162
	0.022038	0.374691	0.396729	0.000471	0.374691	0.375162	0.771891
	0.020399	0.366991	0.38739	0.000436	0.366991	0.367426	0.754816
	0.010499	0.639605	0.650104	0.000224	0.639605	0.639829	1.289933
	0.020215	0.359205	0.37942	0.000432	0.359205	0.359636	0.739056
	0.008371	0.901973	0.910344	0.000179	0.901973	0.902152	1.812496
	0.00596	1.191433	1.197393	0.000127	1.191433	1.19156	2.388953
	0.005006	1.578813	1.583819	0.000107	1.578813	1.57892	3.162739

Abbreviations: HQ, health hazard; HI, health index

**Figure 6:**
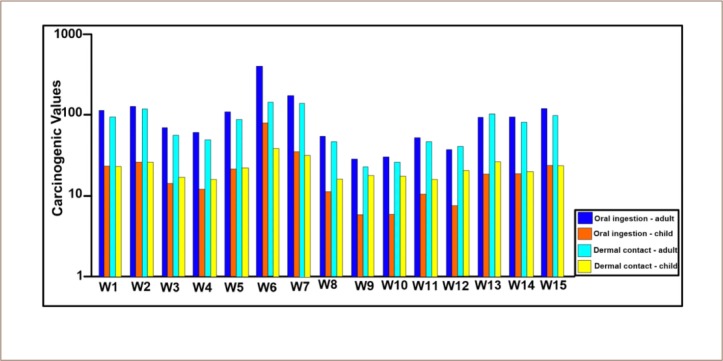
Total non-carcinogenic health index of heavy metals through oral and dermal contact in water samples from Ikenne Abbreviation: W, sampling point

Cadmium, Pb and As present non-carcinogenic health risks *(Table 7)* in both adults and children since the values are above the threshold of 1 recommended by the United States Environmental Protection Agency (USEPA) for non-carcinogenic health diseases, but the effects are greater for children, as revealed by the results in Figure 6.^3^

**Table 7:** Non-Carcinogenic Health Hazard for Adults and Children through Oral Ingestion and Dermal Contact with Groundwater

	**Oral Ingestion**				**Dermal**				**Health Hazard**

	**HQ Cd**	**HQ As**	**HQ Pb**	**HI**	**HQ Cd**	**HQ As**	**HQ Pb**	**HI**	
		
Adults	34.88	0.0009	73.83	108.7	69.75	1.00E-05	20.51	90.3	198.97
	42.51	0.0008	79.89	122.4	85.02	1.00E-05	22.2	107	229.61
	19.63	0.0015	47.14	66.76	39.25	1.00E-05	13.1	52.4	119.11
	17.74	0.0016	39.25	56.98	35.47	1.00E-05	10.91	46.4	103.36
	31.81	0.001	71.37	103.2	63.62	1.00E-05	19.83	83.5	186.63
	17.44	0.0017	363	380.4	34.88	1.00E-05	100.8	136	516.14
	49.15	0.0007	115.4	164.5	98.3	1.00E-05	32.05	130	294.87
	17.3	0.0017	34.6	51.91	34.6	1.00E-05	9.62	44.2	96.13
	8.24	0.0036	18.9	27.14	16.47	1.00E-05	5.25	21.7	48.87
	9.77	0.0033	18.51	28.29	19.54	1.00E-05	5.14	24.7	52.98
	17.6	0.0017	32.25	49.85	35.19	1.00E-05	8.96	44.2	94
	17.07	0.0033	18.12	35.2	34.14	1.00E-05	5.04	39.2	74.39
	42	0.0014	45.48	87.48	83.99	1.00E-05	12.64	96.6	184.11
	29.36	0.001	60.08	89.44	58.72	1.00E-05	16.69	75.4	164.85
	34.63	0.0009	79.61	114.2	69.25	1.00E-05	22.12	91.4	205.6
Children	6.98	9.00E-04	14.77	21.75	13.95	2.854	4.93	21.74	43.48
	8.51	8.00E-04	15.98	24.49	17.01	2.447	5.33	24.79	49.27
	3.93	0.002	9.43	13.36	7.85	5.118	3.15	16.12	29.48
	3.55	0.002	7.85	11.4	7.1	5.461	2.62	15.19	26.59
	6.37	0.001	14.28	20.64	12.73	3.355	4.76	20.85	41.49
	3.49	0.002	72.6	76.09	6.98	5.657	24.2	36.84	112.9
	9.83	7.00E-04	23.08	32.91	19.66	2.153	7.7	29.51	62.42
	3.46	0.002	6.92	10.39	6.92	5.858	2.31	15.1	25.49
	1.65	0.004	3.78	5.44	3.3	12.2	1.26	16.77	22.21
	1.96	0.003	3.71	5.67	3.91	11.29	1.24	16.46	22.12
	3.52	0.002	6.45	9.98	7.04	5.811	2.15	15.01	24.99
	3.42	0.003	3.63	7.05	6.83	11.19	1.21	19.25	26.3
	8.4	0.001	9.1	17.5	16.8	4.634	3.04	24.48	41.98
	5.88	0.001	12.02	17.89	11.75	3.299	4.01	19.06	36.95
	6.93	9.00E-04	15.93	22.85	13.85	2.771	5.31	21.94	44.79

Abbreviations: HQ, health hazard; HI, health index

## Conclusions

Analysis of water quality in the study area illustrates the nature of pollutants found in groundwater as a result of continuous random dumping of hazardous waste and discharge of effluents by agriculture and municipal waste in the community and surrounding environment. The results of this study suggest that groundwater close to the study area is no longer potable for drinking due to the moderate-abundant amounts of carbonate, which causes water hardness, and could lead to cosmetic and aesthetic effects on taste, odor and color. There is also an elevated risk for carcinogenic and non-carcinogenic diseases for adults and children in the area due to high occurrence of Pb, As and Cd found in the water. It is therefore recommended that the municipal government distribute waste disposal bags and establish a waste collection service to reduce indiscriminate dumping of refuse in the area. In addition, a task force should be formed in the community to enforce existing waste disposal and water protection laws. Construction of a centralized, deep, double-cased well is recommended to ensure an uncontaminated water supply for drinking and domestic purposes in the study area.
