# Supplementary material for: Health Risk Assessment of Heavy Metal Pollution in Groundwater Around an Exposed Dumpsite in Southwestern Nigeria
Source: J Health Pollut. 2019 Dec 6;9(24):191210. doi: 10.5696/2156-9614-9.24.191210 (PMC6905142; doi:10.5696/2156-9614-9.24.191210)
Supplement: Supplementary file 1 [file Laniyan_supplemental_material.docx]

**Supplemental Material**

**Bivariate correlation of physicochemical parameters in groundwater of the study area**

|  | **Temp** | **Salinity** | **TDS** | **pH** | **EC** | **SO_4_** | **N_O3_** | **Cl** | **CO_3_** | **HCO_3_** | **Ca** | **Mg** | **Na** | **K** | **Cu** | **Cd** | **Fe** | **As** | **Pb** | **Zn** |
| --- | --- | --- | --- | --- | --- | --- | --- | --- | --- | --- | --- | --- | --- | --- | --- | --- | --- | --- | --- | --- |
| **Temp** | 1 |  | | |  |  |  |  |  |  |  |  |  |  |  |  |  | |  |  |
| **Salinity** | 0.455 | 1 |  | |  |  |  |  |  |  |  |  |  |  |  |  |  |  |  |  |
| **TDS** | 0.242 | **0.634*** | 1 |  |  |  |  |  |  |  |  |  |  |  |  |  |  |  |  |  |
| **pH** | 0.266 | 0.239 | **0.915** | 1 |  |  |  |  |  |  |  |  |  |  |  |  |  |  |  |  |
| **EC** | 0.137 | 0.486 | **0.676**** | 0.264 | 1 |  |  |  |  |  |  |  |  |  |  |  |  | |  |  |
| **SO_4_** | 0.310 | **0.516** | **0.911** | 0.397 | 0.772 | 1 |  |  |  |  |  |  |  |  |  |  |  |  |  |  |
| **NO_3_** | -0.405 | 0.420 | **0.994** | **0.621** | 0.440 | **0.650**** | 1 |  |  |  |  |  |  |  |  |  |  |  |  |  |
| **Cl** | **0.998** | **0.597** | **0.761** | 0.365 | **0.666** | 0.258 | 0.454 | 1 |  |  |  |  |  |  |  |  |  |  |  |  |
| **CO_3_** | **0.576** | 0.222 | **0.899** | 0.152 | 0.352 | **0.606** | 0.418 | **0.664** | 1 |  |  |  |  |  |  |  |  |  |  |  |
| **HCO_3_** | 0.411 | **0.501** | **0.874** | 0.304 | 0.346 | 0.386 | **0.517** | 0.307 | **0.907*** | 1 |  |  |  |  |  |  |  |  |  |  |
| **Ca** | **0.708** | **0.621** | 0.266 | **0.873** | 0.283 | **0.838** | 0.254 | 0.157 | 0.293 | 0.360 | 1 |  |  |  |  |  |  |  |  |  |
| **Mg** | **0.661**** | **0.520** | 0.322 | 0.219 | 0.165 | **0.828** | **0.761** | **0.970** | **0.863** | **0.844** | **0.565** | 1 |  |  |  |  |  |  |  |  |
| **Na** | 0.087 | **0.603** | **0.507** | 0.460 | **0.499** | **0.550** | 0.073 | **0.519** | 0.166 | 0.161 | 0.091 | 0.344 | 1 |  |  |  |  |  |  |  |
| **K** | **0.949** | 0.480 | **0.754** | **0.564** | **0.513** | **0.797** | 0.335 | 0.427 | **1.000** | **0.782** | 0.114 | **0.797** | 0.057 | 1 |  |  |  |  |  |  |
| **Cu** | **0.820** | **0.763** | **0.552** | **0.685** | 0.424 | **-0.627*** | **0.859** | 0.395 | 0.125 | 0.051 | **0.544*** | **0.605** | **0.839** | **0.925** | 1 |  |  |  |  |  |
| **Cd** | **-0.683**** | -0.490 | -0.443 | **0.541** | **-0.545*** | **0.953** | **0.940** | 0.180 | 0.456 | 0.498 | 0.050 | 0.097 | **0.853** | 0.090 | **0.820** | 1 |  |  |  |  |
| **Fe** | 0.492 | 0.072 | 0.427 | **0.608** | **0.536** | 0.016 | 0.435 | 0.063 | **0.935** | **0.609** | **0.904** | **0.788** | **0.672** | 0.372 | 0.009 | 0.108 | 1 |  |  |  |
| **As** | **0.866** | 0.287 | 0.311 | 0.179 | **0.595** | **0.608** | 0.353 | 0.450 | **0.836** | **0.962** | 0.336 | 0.477 | **0.862** | 0.260 | 0.342 | **0.855** | 0.203 | 1 |  |  |
| **Pb** | **0.978** | 0.193 | 0.013 | 0.149 | **0.925** | **0.665** | **0.554** | **0.700** | **0.734** | **0.663** | 0.297 | **0.971** | 0.126 | **0.666** | **0.721** | **0.544** | **0.613** | **0.633** | 1 |  |
| **Zn** | 0.087 | **0.603** | **0.507** | 0.460 | 0.499 | **0.550** | 0.073 | **0.519** | 0.166 | 0.161 | 0.091 | 0.344 | **1.000**** | **0.501** | **0.839** | **0.853** | **0.672** | **0.862** | 0.126 | 1 |

*Correlation is significant at the 0.05 level (2-tailed); ** Correlation is significant at the 0.01 level (2-tailed); Abbreviation: Temp., temperature.
